# Comparison of serum levels of copper and zinc among multiple sclerosis patients and control group

**Published:** 2013

**Authors:** Behnaz Sedighi, Hossein Ali Ebrahimi, Ali Akbar Haghdoost, Marzie Abotorabi

**Affiliations:** 1 Associate Professor, Department of Neurology AND Kerman Neurology Research Center, Kerman University of Medical Sciences, Kerman, Iran; 2 Professor, Department of Neurology, Kerman University of Medical Sciences, Kerman, Iran; 3 Professor, Department of Epidemiology, Kerman University of Medical Sciences, Kerman, Iran; 4 Resident, Department of Neurology, Kerman University of Medical Sciences, Kerman, Iran

**Keywords:** Copper, Zinc, Multiple Sclerosis

## Abstract

**Background:**

There have been several studies done on the role of metals in the occurrence of multiple sclerosis (MS) disease, but their roles have not been confirmed yet. Because of the lack of information on this issue, this study compared the serum level of copper and zinc in MS patients with their levels in a control group.

**Methods:**

This was an analytical, cross-sectional study conducted in Kerman (a medium size city), Iran. We assessed the serum level of copper and zinc in 58 MS patients and 39 healthy individuals, who were selected from the relatives of cases and matched for age and sex.

**Results:**

The average serum level of Copper in cases and controls were 93.7 and 88.9 ml/dl, respectively. The corresponding numbers for Zinc were 36.7 and 40.9 ml/dl, respectively. There was no significant difference between the two groups (copper: P = 0.459; zinc: P = 0.249).

**Conclusion:**

The groups were matched for age, sex, and family. However, we did not find a considerable difference between the level of these metals in MS patients and controls.

## Introduction

MS is a chronic demyelinating disease of the central nervous system; caused by an immune reaction that happens in a susceptible genetic condition.^1–3^ The trigger of immunity responses is totally unknown, but some possible factors (such as vitamin D deficiency, previous infection by Epstein-Barr virus (EBV), and being in contact with metals) have been implicated.^4–6^

Zinc (Zn) has a key role in regulation of the immune system. For instance, Zn is involved in releasing tumor necrosis factor alpha (TNFα), which activates the immune system.^7, 8^ It was shown that even a mild Zn deficiency can weaken the function of the immune system.^9^

Cooper (Cu) is used in the synthesis of myelin; thus, the deficiency may potentially cause myelinopathy.^4, 10^ Furthermore, its influence on auto immune diseases through the catalyzation of prostaglandins (anti-inflammatory drugs) has been known. In addition, it is documented that there is an interaction between Zn and Cu. This means that a high level of Zn could be a reason for Cu and iron deficiency. Moreover, Cu and some other elements, such as cadmium and mercury, may compete with Zn.^4, 11^

There are many questions about the role of metals in the pathogenesis of MS. Some studies showed that an increase in Cu and a decrease in Zn might stimulate the immune system toward MS.^12^ However, some other studies highlighted the etiological role of magnesium and Cu.^13^

Because of these controversies, we explored possible associations between MS and the serum level of Zn and Cu in Kerman, Iran. Kerman is located in the south-east of Iran with a mostly dry climate. MS is more or less common in this part of Iran (with a prevalence of 31.5 per 100,000 population for the whole province, and 57 in the center the province).

We have compared Cu and Zn in new cases of MS in the Kerman province with their first degree relatives (to eliminate the confounding factor of genetics), and investigated the effects of the two elements on multiple sclerosis.

## Materials and Methods

The study was an analytical, cross-sectional study. Newly diagnosed MS cases, 59 patients diagnosed based on the McDonald criteria, were recruited. These patients were referred to a neurology referral hospital, their disease was confirmed, and they were registered in the list of the MS society in Kerman province. We actively approached the family members of our cases to find an age and sex adjusted control among the first relatives.

We obtained informed written consents from both cases and controls, before obtaining data and taking blood samples.

Our inclusion criteria for both groups were: 1. living in Kerman province in the previous 10 years; 2. not received any types of Zn and Cu supplements; 3. not received any types of corticosteroids in the previous month; and 4. not contracted a type of diseases which might have interaction with Cu and Zn in the last year.

Having applied the above inclusion criteria, we managed to collect 58 case and 39 control subjects. Most of dropped outs were from the controls; 17 individuals did not accept to participate even after the main objectives of the study were explained to them in detail.

From eligible cases and controls, 10 ml of blood was taken, and transferred to a research laboratory in the Kerman University of Medical Sciences. The total levels (free and carrier bounded) of Zn and Cu of serums were measured using an atomic absorption spectrophotometer (Shimadzu AA-670).^14^

After collecting data, subjects were categorized based on their serum levels for Cu and Zn into normal or abnormal. The used cut off point for Zn was 70 micrograms per deciliter, and for Cu it was between 70 to 155 micrograms per deciliter.^9^

The mean Zn and Cu concentrations and the frequency of abnormal concentrations were compared in cases and controls using Student's t-test, chi-square, or Fisher's exact test.

## Results

10 of 58 MS cases and 9 of 39 controls were male. All patients were relapsing-remitting MS patients; the average time passed from the diagnosis was 58 days.

**Figure 1:**
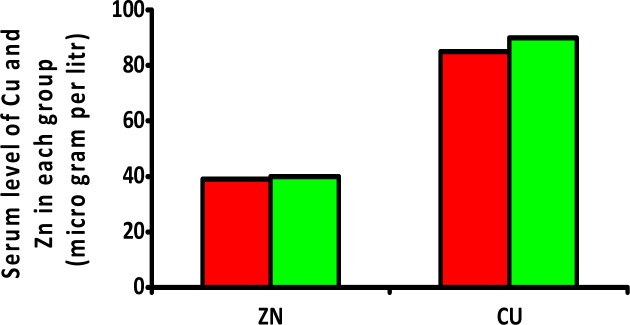
The mean serum level of Cu and Zn in cases and controls

The mean of Cu and Zn serum in cases and controls are shown in Figure 1. In both groups, the serum level of Cu was greater, but the level of these two elements were comparable in cases and controls (Cu: P = 0.46, Zn: P = 0.25).

The frequency of abnormal Cu in cases was 12 (21.1%) and the corresponding frequency in controls was 10 (26.3%) (P = 0.57).

The frequency of abnormal Zn among cases and controls was 53 (93%) and 35 (97.2%), respectively (P = 0.38)

Overall, 22 (23.2%) of all subjects (case and controls together) had abnormal Cu, and 88 (94.6%) had decreased level of Zn.

## Discussion

Based on our findings, the level of Cu and Zn in serum of MS cases and controls were more or less comparable; although, more than 90% of subjects in both groups had low level of Zn.

Our findings did not show any association between the serum concentration of Zn and Cu, and MS. These results were compatible with the findings of the study by Geeta. However, the study found that Cu in the diet of patients was less than the recommended quantity.^15^ Smith et al. have reported that Zn concentrations were comparable in patient and control groups, but Cu was lower in patients. This study suggested that impaired Cu and Zn homeostasis may be a cause of MS disease.^16^

However, there are a few studies which reported that the serum level of Zn and Cu in MS patients was less than that in the controls.^17–19^ One possible reason for lower levels in patients might be the malabsorption of these elements in MS patients.^20^ Based on clinical experiences, Johnson believed that the gradual decrease in Zn and increase in Cu is one of the triggers of MS attacks.^12^ Moreover, the co-occurrence of Wilson disease and MS has been reported.^20^ A toxic level of Cu may trigger immunological reactions against myelin in the brain.^21^ Finally, a study on individuals with Down's syndrome showed that deficiency of micronutrients such as magnesium, Cu, and cobalt, and toxic levels of heavy metals (such as manganese, lead, and iron) might be Involved in the aetiology of MS disease.^13^

We selected the control group from the first relatives of patients to eliminate the possible confounding effects of genetic factors, which may play a significant role in the aetiology of this disease.^1–3^ In a similar genetic background it was easier to examine the possible effect of Cu and Zn as environmental factors. Some studies had shown a possible relationship between Cu and Zn levels, and MS disease. However, the observed association might be due to the effect of corticosteroid treatments.^16, 18^ To minimize these effects in the present study, we selected newly diagnosed patients. Therefore, the average duration from the time of diagnosis to the beginning of our study was only 58 days. In addition, to minimize the effect of corticosteroid, samples were selected from those who had not received these treatments in the last month.

In comparison with similar studies, in the current study we selected our samples in a way that minimized the impact of confounding factors. Therefore, the lack of association between Zn and Cu, and MS might be supported by our findings.

In this study, it was observed that 94.6% of all subjects (93% of MS patients and 97.2% of the controls) had a low level of Zn. Zn is an element that is highly influenced by environmental and nutritional status. One of the causes of Zn deficiency is contact with some elements that might compete with Zn, such as cadmium, Mercury, and Cu.^4, 11^ Moreover, Previous studies on the Copper Industries of Kerman showed that the serum level of Zn is lower in these groups.^14^ Consequently, it seems that a low level of Zn in both groups might be because of living in the same environment with a high level of exposure to Cu. One of the limitations in this study was that we did not explore the diet of our subjects. We suggest that future studies consider the diet of subjects. The serum level of Cu and Zn also need to be compared in MS patients who have a background of working in Copper Industries and those lacking this background. We also suggest that a similar study be conducted in the neighboring provinces which do not have copper industries.

## Conclusion

We came to the conclusion that if we eliminated the factors contributing to Zn and Cu homeostasis, there would be no difference in Zn and Cu levels of MS patients compared with the control group which are matched in terms of genetics and environmental condition.
